# Effect of antithyroid antibodies on women with recurrent miscarriage: A meta‐analysis

**DOI:** 10.1111/aji.13238

**Published:** 2020-04-11

**Authors:** Jilai Xie, Lihong Jiang, Annapurna Sadhukhan, Songqing Yang, Qiuping Yao, Ping Zhou, Jinpeng Rao, Min Jin

**Affiliations:** ^1^ Second Affiliated Hospital School of Medicine Zhejiang University Hangzhou China; ^2^ Taizhou Women and Children’s Hospital Affiliated to Wenzhou Medical University Taizhou China

**Keywords:** autoimmunity, LT4 treatment, meta‐analysis, recurrent miscarriage, thyroid antibody

## Abstract

**Problem:**

The effect of thyroid autoimmunity (TAI) on the prevalence of recurrent miscarriage (RM) is highly debatable. No meta‐analysis has been published in the past decade to investigate the impact of TAI on women with RM.

**Method of Study:**

Systemic literature search was conducted on PubMed, Embase, Cochrane, and Web of Science databases. English language literatures published between 1993 and 2019 were selected. We assessed the relationship between the prevalence of RM and thyroid peroxidase antibodies (TPO‐Ab) or antithyroid antibodies (ATA) and evaluated the thyroid‐stimulating hormone (TSH) level in TPO‐Ab‐positive women with RM. We also observed the treatment effect with levothyroxine (LT4) for RM. Review Manager 5.3 software was used to obtain the pooled odds ratios (OR).

**Results:**

Analysis of 22 eligible studies revealed significant association between TPO‐Ab and the prevalence of RM (OR = 1.85; 95% CI, 1.38 to 2.49; *P* < .001)(n ≥ 3), (OR = 1.82; 95% CI, 1.13 to 2.92; *P* = .01) (n ≥ 3). Women with ATA + had higher risk of RM (OR = 2.36; 95% CI, 1.71 to 3.25; *P* < .00001)(n ≥ 3), (OR = 2.34; 95% CI, 1.70 to 3.22; *P* < .00001)(n ≥ 2). RM women with TPO‐Ab had higher TSH level when compared with those negative for TPO‐Ab (random‐effect SMD = 0.60; 95% CI, 0.31 to 0.90; *P* < .0001). We also found beneficial effects of LT4 supplementation on the outcome of live birth rate (LBR) among pregnant women with TPO‐Ab (OR = 3.04; 95% CI, 0.69 to 13.36; *P* = .14).

**Conclusion:**

The presence of serum antithyroid antibodies does harms to women and can even lead to recurrent miscarriage; LT4 treatment may have beneficial to RM women.

## Graphical abstract

This study reveals the association between thyroid autoimmunity and TSH level, the prevalence of RM. And LT4 supplementation may effectively increase live birth rate.

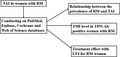

## 1 INTRODUCTION

Thyroid disease is one of the most frequent endocrine conditions in women of childbearing age.^1^ The most common cause of thyroid dysfunction is thyroid autoimmunity (TAI).^2^ TAI is defined as the presence of antithyroid antibodies (ATA), specifically thyroid peroxidase antibodies (TPO‐Ab) and/or thyroglobulin antibodies (Tg‐Ab).^3^ With a prevalence of 5%‐20%, TAI is the most common autoimmune condition in women of reproductive age.^4^

Thyroid hormones play a role in menstrual cycle and in achieving fertility as they affect the actions of follicle‐stimulating hormone and luteinizing hormone on steroid biosynthesis by specific T3 sites on oocytes.^5^ Thyroid autoimmunity has been found to be related to subclinical hypothyroidism (SCH),^6^ which is defined as high levels of serum thyroid‐stimulating hormone (TSH) despite normal levels of serum free thyroxine (FT4).^7^ Both thyroid dysfunction and thyroid autoimmunity are known to cause adverse pregnancy outcomes during all trimesters of pregnancy.^8^ Nevertheless, recent evidence shows an association between euthyroid women with thyroid autoimmunity and poor obstetric outcomes. The presence of ATA, particularly TPO‐Ab, has been associated with miscarriage, preterm birth, and post‐partum thyroid disease.^9^, ^10^, ^11^

Recurrent miscarriage (RM) has previously been defined as three or more pregnancy losses^12^ which affects 1% of couples. However, in recent years, more guidelines have redefined it as two or more pregnancy losses^13^, ^14^ which affects < 5% of couples.^15^ RM places a severe physical, emotional, and financial burden on many families and our communities. An effective management and treatment is necessary. A higher prevalence of TPO‐Ab in women with RM has been found in several studies, varying from 19% to 36%.^16^, ^17^, ^18^

A majority of women with TAI have detectable antibodies such as TPO‐Ab and sometimes TG‐Ab, whereas few have TSH receptor antibodies.^19^ The prevalence of TPO‐Ab ranges for 8% to 14% in women of reproductive age.^20^ The prevalence of positive‐TPO‐Ab among pregnant women in countries with good iodine supply has been reported between 5.1^21^, ^22^ and 12.4%.^23^ The effect of thyroid autoimmunity especially TPO‐Ab on the clinical outcome of RM is highly debated. In recent years, no new meta‐analysis has been published to reveal the relationship between RM and TPO‐Ab or ATA.^24^ And the expanded definition of RM affects differential amounts of women of childbearing age. In the increasing affected women, the assay of TPO‐AB or ATA whether can effectively predict RM, it need more data.

In recent researches, TPO‐Ab is associated with unexplained RM and these women may benefit from treatment with levothyroxine (LT4)^25^, ^26^ with decreasing TPO‐Ab levels after 2‐3 months treatment.^26^ But in other studies, euthyroid women with TPO‐Ab undergo a treatment with levothyroxine, compared with no levothyroxine treatment, did not reduce miscarriage rate or increase live birth rate.^27^, ^28^ It is therefore possible that patients with RM and TPO‐Ab benefit from the substitution of thyroid hormones in terms of a lower rate of miscarriage, but currently there are no data specifically on patients with RM.

Currently, the effect of thyroid autoimmunity by itself on the prevalence of RM has not been established yet. Moreover, there was only one guideline(reference)^15^ with regard to the need of treatment for women with RM, but there is no management for thyroid autoimmune in the guideline, more information is needed for developing a new guideline. Combining data on this controversial issue might reveal useful information for the counseling and management of euthyroid TPO‐Ab + or ATA + women with RM. This study aims to conduct a systematic review and meta‐analysis to gain insight into the clinical significance of TAI in women with RM.

## 2 METHODS

### 2.1 Literature search strategy

The meta‐analysis was conducted according to the Preferred Reporting Items for Systematic Reviews and Meta‐Analyses (PRISMA) 2009 guidelines.^22^ This systematic review was restricted to published research articles that compared the prevalence of RM in women positive for TPO‐Ab or ATA with that observed in women negative for thyroid autoantibodies. We also compared the relationship between RM and TPO‐Ab or ATA in different definition of RM. A literature search was conducted on PubMed, Embase, Cochrane, and Web of Science using combinations of the medical subject heading terms: ('thyroid autoimmunity' or 'thyroid autoantibody' or 'thyroid gland' or 'thyroid') AND ('recurrent pregnancy loss' or 'recurrent spontaneous abortion' or 'recurrent miscarriage' or 'habitual abortion' or 'recurrent spontaneous miscarriage' or 'habitual abortion'). The main search was conducted from inception through to December 2019 and was restricted to English literature. All relevant articles were retrieved, and additional reports were chosen from their reference lists. Review articles published on thyroid autoimmunity were also consulted for further pertinent studies. Unpublished studies were not identified.

### 2.2 Study selection

Only English language literatures were included in our study. Published cohort or case‐control studies (retrospective or prospective) that described at least 10 patients were eligible for inclusion. Studies were excluded if only TAI‐positive women were reported without a control group of TAI‐negative women. Studies were excluded if women were known to have overt biochemical hypothyroidism or hyperthyroidism history or were receiving any treatment for thyroid dysfunction.

The quality of the included studies was assessed by the Newcastle–Ottawa scale, a validated modality for assessing observational and non‐randomized studies.^29^ The scale uses a score system based on three major criteria: selection of participants, comparability of study groups, and completeness of follow‐up. A quantitative appraisal of overall quality of each observational study was obtained, and scores ranged from 6 to 8 (Table 1).

**Table 1:** Characteristics of the studies included in the quantitative analysis

First author	Year	Study type	Participants	Consecutive abortions	Hormone levels	Patients	Controls	Outcome measures	Quality features^a^	Intervention
Bagis	2001	Prospective study	876 women	≥3 or ≥ 2	TPO‐Ab, Tg‐Ab: Chemiluminescent enzyme immunometric assay method. Positive: >35 IU/mL for TPO‐Ab and ＞40 IU/mL for Tg‐Ab. TSH: microparticle enzyme immunoassay method, normal range: 0.3‐4.0 μU/mL	81 women positive for TPO‐Ab	795 women negative for TPO‐Ab	RM	8	–
Bellver	2008	Prospective study	30 women with RM, 32 healthy controls.	≥2	Anti‐TPO and anti‐TG were studied with a two‐site immunoluminometric assay. Normal range anti‐TPO: <25 UI/mL and anti‐TG: <100 UI/mL	4 women positive for TPO‐Ab	58 women negative for TPO‐Ab	RM	6	–
Bliddal	2019	Cohort study	825 women with RM	≥3	TPO‐Ab was measured by the automated Kryptor immunofluorescence assay. TPO‐Ab positivity: ≥60 kIU/L	139 women positive for TPO‐Ab	686 women negative for TPO‐Ab	LBR	7	T4 treatment. Four of the women were treated with Euthyrox (Merck), one woman with Levaxin (Takeda) (75 μg/d), and the rest with Eltroxin (Aspen)
Bussen	1995	Case‐control study	22 euthyroid non‐pregnant habitual aborters and 22 multigravidae without endocrine dysfunction served as controls.	≥3	TPO‐Ab and Tg‐Ab were assayed using enzyme‐linked immunosorbent assay kits. positive: TPO‐Ab, Tg‐Ab or both antibodies(＞100 IU/mL).	6 TPO‐Ab+, 9 ATA+	38 TPO‐Ab−, 35 ATA−	RM	8	–
Bussen	1997	Case‐control study	28 non‐pregnant women with a history of RM and 28 multigravidae without endocrine dysfunctions	≥3	TPO‐Ab and TG‐Ab were assayed using ELISA kits. A positive result in both assays was defined as titers >100 IU/mL	7 TPO‐Ab+, 13 ATA+	49 TPO‐Ab−, 43 ATA−	RM	7	–
Cueva	2018	Cohort study	74 women with recurrent early pregnancy loss who were euthyroid or had subclinical hypothyroidism	≥2	Presence of maternal antithyroid antibodies was defined as anti‐TPO antibodies > 4 IU/mL or anti‐Tg antibodies > 9 IU/mL	13 women positive for TPO‐Ab	61 women negative for TPO‐Ab	TSH level	8	–
Dendrinos	2000	Case‐control study	30 euthyroid women with RSM aged 25‐37 y were compared with 15 matched fertile controls	≥3	Thyroid peroxidase(TPO) and thyroglobulin antibodies were tested with a chemiluminescence immunoassay. The normal range for this assay was < 2 IU/mL	13 ATA+	31 ATA−	RM	6	–
Dobson	2018	Retrospective cohort review	242 patients with RM	≥3	TPO‐Ab not defined	12 women positive for TPO‐Ab	230 women negative for TPO‐Ab	LBR	6	thyroxine, Unknown dosage
Esplin	1998	Case‐control study	74 RM and 75 healthy, fertile control	≥3	Levels of IgG anti‐Tg and IgG anti‐TPO were measured by means of radioimmunoassay kits. Normal range 0.36‐12 units/mL	71 TPO‐Ab+, 82 ATA+	78 TPO‐Ab−, 67 ATA−	RSM	8	–
Iravani	2008	Case‐control study	A total of 641 RM patients and 269 healthy controls were included	≥3	TG‐Ab, TPO‐Ab were measured with ELISA method. Positive: Tg‐Ab >125 IU/mL, TPO‐Ab >40 IU/mL. TSH was tested by immunoradiometric assay, reference ranges of 0.4‐4 mIU/L	145 women positive for TPO‐Ab	765 women negative for TPO‐Ab	RSM, TSH level	6	–
Junhao Yan	2012	Cohort study	496 women with unexplained RM and a control group of 220 women with a known cause for RM were included in the study	≥3	ELISA method. Positive: TPO‐Ab was > 50 U/mL	34 women positive for TPO‐Ab	330 women negative for TPO‐Ab	LBR	8	Some patients were given empirical thyroxine therapy with 50 mg of thyroxine, whereas others were given no treatment at all
KAIDER	1999	Case‐control study	591 patients with recurrent pregnancy loss and 100 normal healthy individuals.	≥3	Serodia gel‐agglutination assays were used to test TPO‐Ab and Tg‐Ab. The positive threshold was					
established by manufacturer as greater than a titer of 1:300.	61 TPO‐Ab+, 85 ATA+	337 TPO‐Ab−, 313 ATA−	RM	7	–					
Kutteh	1999	Retrospective, two‐centered study	700 women with a history of RM and 200 healthy, reproductive‐aged female controls	≥2	TPO‐Ab and TG‐Ab were assayed using commercial ELISA test kits. Negative: ≤67 IU/mL for thyroglobulin and ≤ 40 IU/mL for thyroid peroxidase	126 TPO‐Ab+, 187 ATA+	774 TPO‐Ab−, 713 ATA−	RM	8	–
Lata	2013	Case‐control study	100 pregnant and 25 non‐pregnant women with a history of RM, 100 pregnant women without a history of RM as healthy controls	≥2	TSH and anti‐TPO is assessed by electro‐chemiluminescence immunoassay. The reference range for the above hormones are as follows: TSH, RR: 0.27‐4.2 μIU/mL and anti‐TPO, RR: <34 IU/mL	49 women positive for TPO‐Ab	151 women negative for TPO‐Ab	RM, TSH level	8	All patients with TPO‐Ab + were treated with 25 μg/L‐T4 and titrated according to TSH at the time of recruitment into the study
Mecacci	2000	Prospective study	29 women with a history of early pregnancy loss and 69 healthy control	≥2	Serum levels of TSH were determined by RIA kit (ICN) (normal range 0.2‐4.0 μU/L).RIA kits (Biocode) were employed for the determination of anti‐TG (normal values ≤ 50 IU/mL) and anti‐TPO (normal values ≤ 10 IU/mL) antibodies	21 ATA+	77 ATA‐	RM, TSH level	7	–
Mosaddegh	2012	Cohort study	900 women who had a history of recurrent pregnancy loss	≥2	Thyroid peroxidase (TPO) was tested with a chemiluminescence immunoassay, and women with anti‐TPO more than 40 UI/mL were treated with levothyroxine after signing inform consent	45 TPO‐Ab+, 39 use LT4	6 TPO‐Ab + women never used Levothyroxine by their own decision	LBR	6	Levothyroxine doses were depended on the levels of anti‐TPO, which were decided by endocrinologist. It was 25‐100 μg every day. Treatment continued with levothyroxine and aspirin till pregnancy happened and these continued during pregnancy until delivery.
Motak‐Pochrzęst	2013	Retrospective study	155 patients with primary RM and 50 control patients were analyzed	≥3	Tg‐Ab and TPO‐Ab were detected using immunoassay ELISA. Titers over 60 IU/mL for anti‐Tg and for anti‐TPO were considered to be positive	42 ATA+	163 ATA−	RM	6	–
Mumusoglu	2015	Retrospective study	515 women of reproductive age	≥2	Radioimmunoassay determined TPO‐Ab. Levels of 80 IU/mL were considered positive for TPO‐Ab	67 women positive for TPO‐Ab	448 women negative for TPO‐Ab	RM	6	–
Pratt	1993	Retrospective study	45 RSM patients and 100 healthy controls	≥3	TPO‐Ab and Tg‐Ab were assayed with Kalibre radioimmunoassay kits. A positive result in both tests was defined as ≥0.3 U/mL	25 TPO‐Ab+, 33 ATA+	120TPO‐Ab−, 112 ATA−	RM	7	–
Roberts	1996	Case‐control study	53 pregnant or non‐pregnant women	≥3	TPO‐Ab, Tg‐Ab were tested by using ELISA kits. Positive: Tg‐Ab > 8 U/mL, TPO‐Ab > 1U/mL	4 ATA+	18 ATA−	RM	8	1
Ticconi	2011	Case‐control study	160 women with RM and 100 healthy women	2 or ≥ 3 pregnancy losses	TG‐Ab and TPO‐Ab were detected using CLIA immunoassay. The sensitivity of the TPO‐Ab test was 25 IU⁄mL	39 women positive for TPO‐Ab	221 women negative for TPO‐Ab	RM	7	–
Vissenberg	2015	Retrospective cohort study	344 euthyroid women with unexplained RM	≥2	TPO‐Ab was measured by a chemiluminescence immunoassay. TPO‐Ab− positivity was defined as TPO‐Ab > 60 kU/L	28 TPO‐Ab− positive women	174 TPO‐Ab− negative women	LBR, TSH level	6	levothyroxine, Unknown dosage

Abbreviations: AI, thyroid autoimmunity; ATA, antithyroid antibody; LBR, live birth rate; RM, recurrent miscarriage; TPO‐Ab, thyroid peroxidase antibody.

^a^ Based on the Newcastle‐Ottawa scale.^29^

### 2.3 Data extraction

Two authors independently evaluated all articles and abstracted data on standardized forms. Information regarding study characteristics (author name, year of publication, study design, sample size), methodology (definition of RM, thyroid autoantibodies and hormone measurement method, threshold and time of measurement, study quality) and characteristics of study groups, and outcome (prevalence of RM, serum TSH levels and live birth rate (LBR)) were extracted. Live birth rate was defined as the number of deliveries that resulted in at least one live born baby.

### 2.4 Statistical analysis

The overall effect of TPO‐Ab or ATA on a binary variable (RM‐prevalence, live birth rate) was assessed by a combined odds ratio (OR). The overall impact of TPO‐Ab on a continuous variable (TSH) was assessed by the difference in means for the two groups of patients; and by using the summary data published in the eligible studies, the results for outcomes were expressed as odds ratios (OR) with 95% confidence intervals (CI).^30^

The inconsistency of studies’ results was measured using Cochrane Q and the *I* ^2^ statistics.^31^ *I* ^2^ value of 0% indicates no observed heterogeneity, *I* ^2^ < 25% shows insignificant heterogeneity, *I* ^2^ values of 25%‐50% shows moderate heterogeneity and *I* ^2^ > 50% indicates significant heterogeneity.^31^ The odds ratios (OR) were combined using a fixed‐effects model when heterogeneity observed among studies was absent to moderate; the DerSimonian & Laird method for a random‐effects model was employed when heterogeneity was high (*I* ^2^ > 50%).^32^, ^33^ All statistical analyses were performed using Review Manager 5.3 software (Cochrane Collaboration); *P* < .05 was considered statistically significant.

Funnel plots, which graph OR on a log scale (effect) against standard error of log‐OR (precision), were generated and visually inspected for asymmetry to determine if the included studies were non‐representative of the body of possible studies on the subject (as could result from small study effect or other biases, such as publication and poor‐quality bias).

## 3 RESULTS

### 3.1 Search results and study characteristics

**Figure 1:**
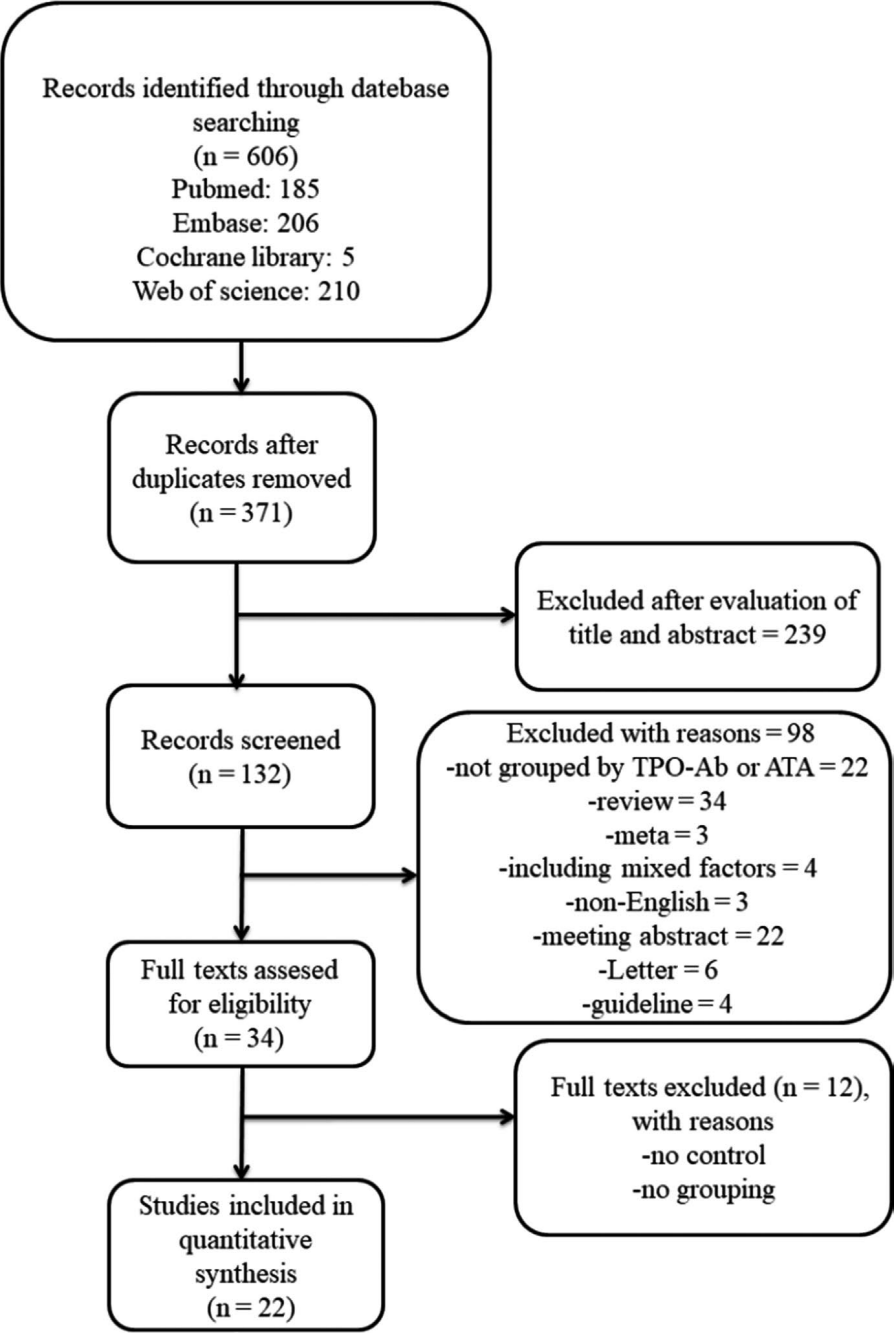
Eligibility of Studies for Inclusion in Meta‐analysis

The process of literature identification and selection of studies is summarized in Figure 1A total of 606 potentially relevant studies were identified through literature search. Out of these, 235 were double citations and 239 were rejected on the basis of title and abstract. The remaining 34 articles were reviewed in full. After evaluation of the full manuscripts, only 22 studies qualified for the final quantitative analysis. The twenty‐two studies included in the systematic review were published between 1993 and 2019. The data from the twenty‐two studies included 952 TPO‐Ab‐positive and 5315 TPO‐Ab‐negative women, 644 ATA‐positive and 1572 ATA‐negative women.

The characteristics of the studies included in the quantitative analysis are summarized in Table 1. Six of these were retrospective cohort studies, three were prospective cohort studies, and four were cohort studies and nine were case‐control studies (Table 1).

### 3.2 Prevalence of RM

**Figure 2:**
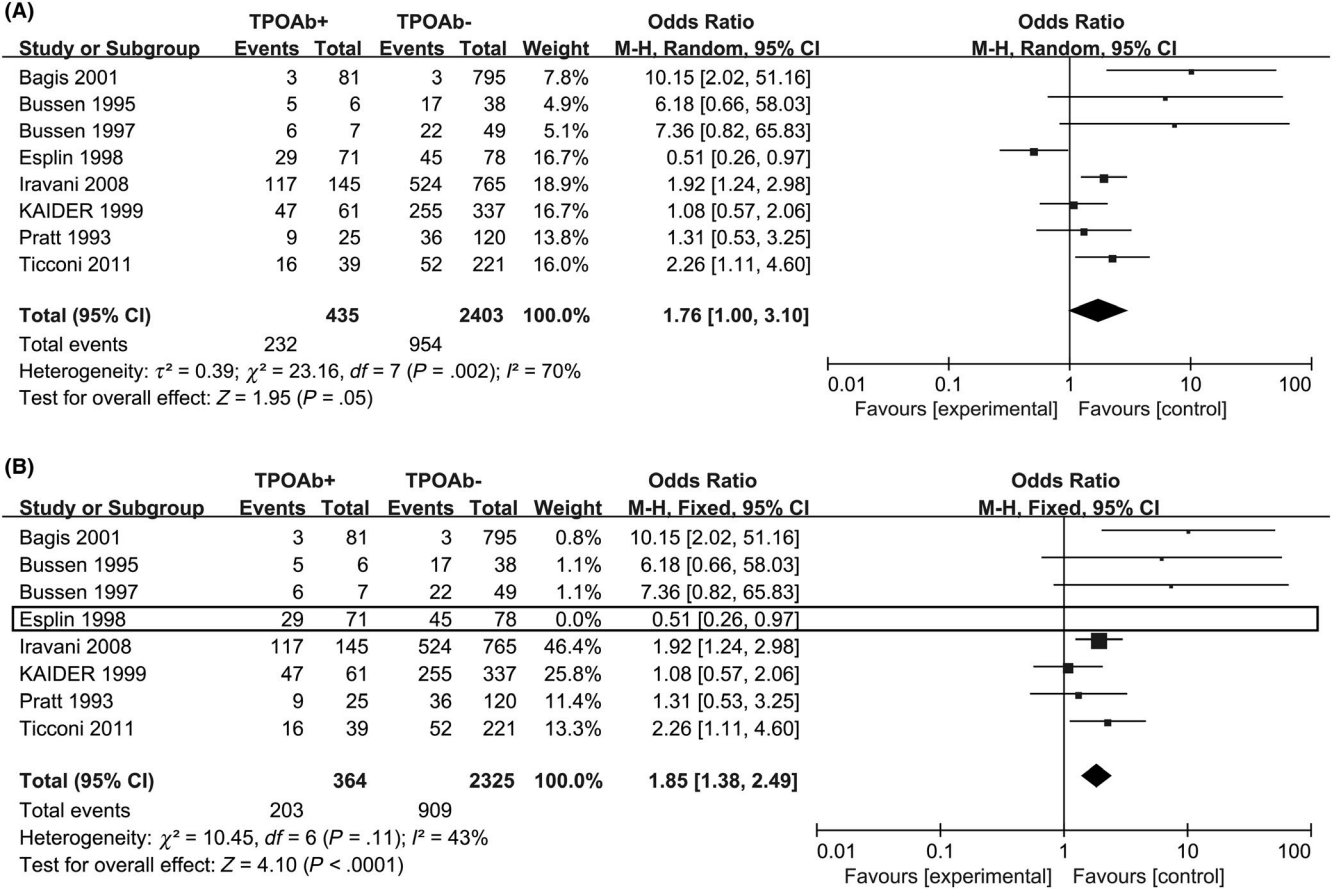
Forest plot of prevalence of RM (n ≥ 3) comparing TPO‐Ab + and TPO‐Ab− women. TPO‐Ab+ = positive for thyroid peroxidase; TPO‐Ab− = negative for thyroid peroxidase

In the women with RM (n ≥ 3), comparing TPO‐Ab‐negative ones, TPO‐Ab‐positive women showed a higher prevalence of RM (random‐effects OR = 1.76; 95% CI, 1.00‐3.10; *P* = .05; Figure 2A). The heterogeneity test result was high (*I* ^2^ = 70%). However, on excluding the study by Esplin,^34^ the degree of heterogeneity declined from high to low (*I* ^2^ = 43%, Figure 2B), indicating that this study was heterogeneous with other studies. Therefore, this study was not included in the following subgroup analysis.

**Figure 3:**
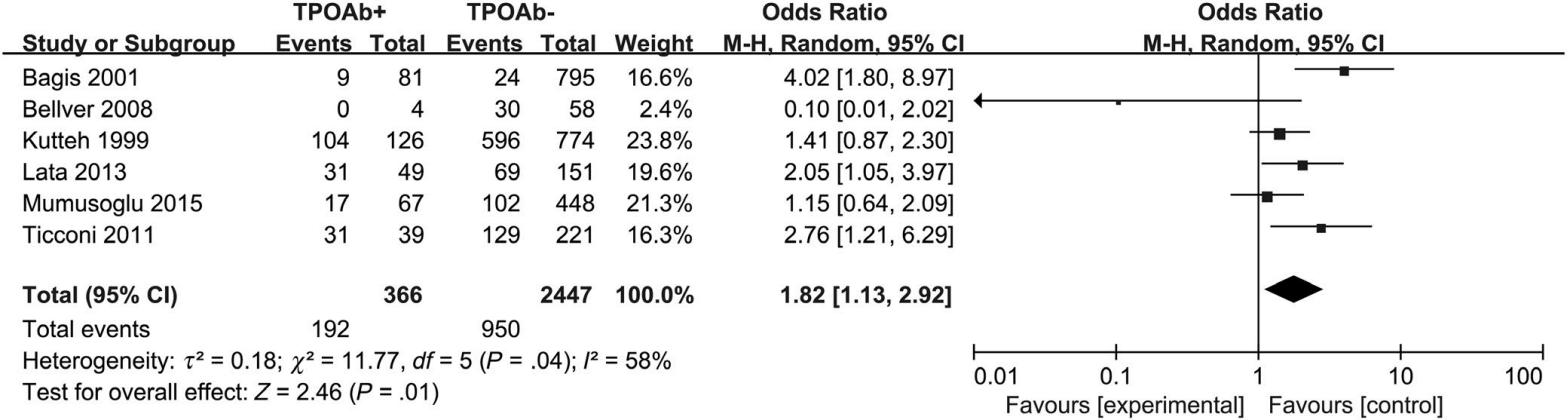
Forest plot of prevalence of RM (n ≥ 2) comparing TPO‐Ab+ and TPO‐Ab− women. TPO‐Ab+ = positive for thyroid peroxidase; TPO‐Ab− = negative for thyroid peroxidase

When we defined RM as ≥ 2 consecutive abortions, in all, 366 TPO‐Ab‐positive pregnancies and 2447 TPO‐Ab‐negative pregnancies were followed up. The result was that 192 aborted in the TPO‐Ab‐positive group, while 950 aborted in the TPO‐Ab‐negative group. Positive‐TPO‐Ab women showed a higher prevalence of RM (random‐effects OR = 1.82; 95% CI, 1.13‐2.92; *P* = .01; *I* ^2^ = 58%; Figure 3).

**Figure 4:**
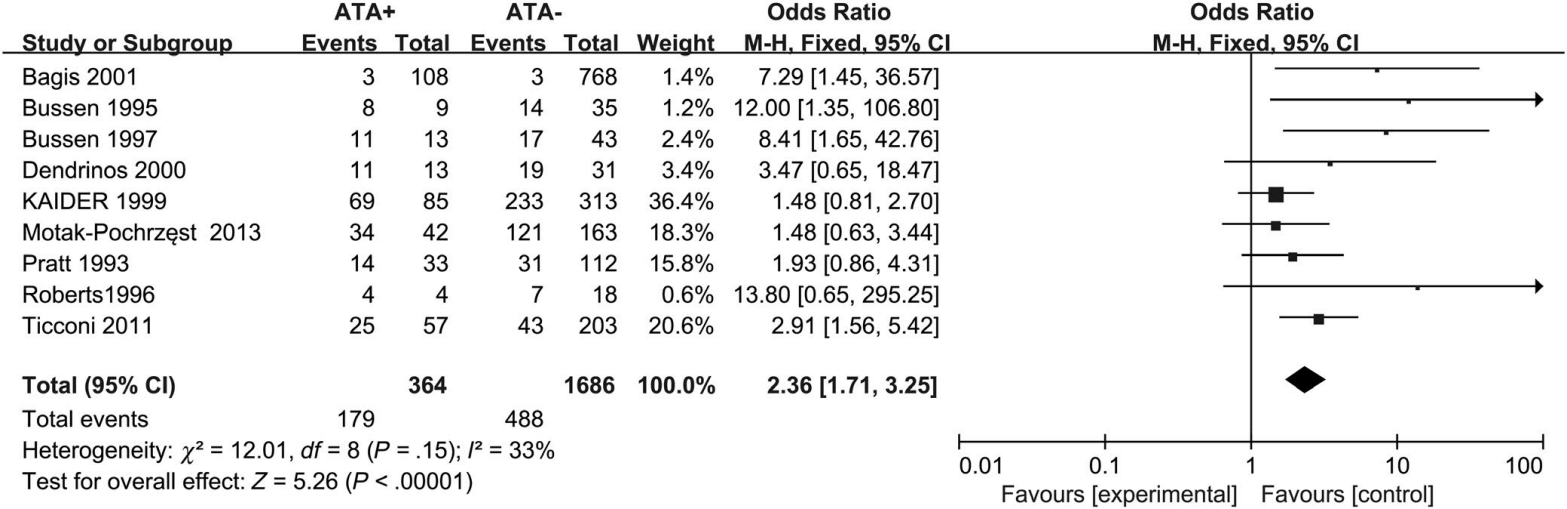
Forest plot of prevalence of RM (n ≥ 3) comparing ATA+ and ATA− women. ATA+ = positive for antithyroid antibodies; ATA− = negative for antithyroid antibodies

**Figure 5:**
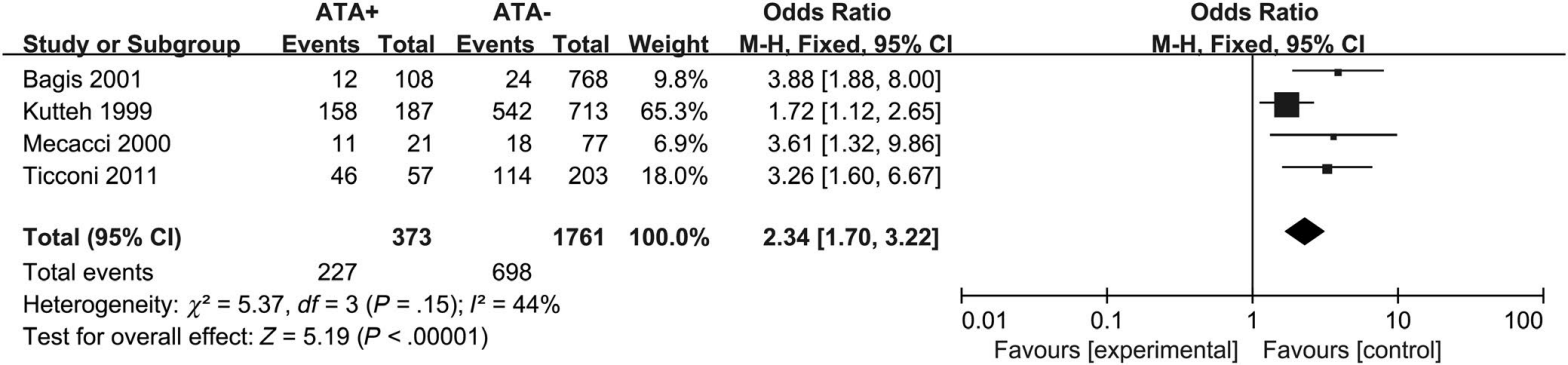
Forest plot of prevalence of RM (n ≥ 2) comparing ATA+ and ATA− women. ATA+ = positive for antithyroid antibodies; ATA− = negative for antithyroid antibodies

Diversity in the methodology applied to measure thyroid autoantibodies was also observed. In twelve studies, TPO‐Ab were detected.^17^, ^34^, ^35^, ^36^, ^37^, ^38^, ^39^, ^40^, ^41^, ^42^, ^43^, ^44^ Both TPO‐Ab and Tg‐Ab were determined in eleven studies.^18^, ^35^, ^36^, ^37^, ^38^, ^39^, ^43^, ^44^, ^45^, ^46^, ^47^ When consecutive abortions ≥ 3, 12 studies showed an increased risk for RM in women with positive ATA compared with negative‐ATA controls (fixed‐effects OR = 2.36; 95% CI, 1.71‐3.25; *P* < .00001; Figure 4), with moderate heterogeneity (*I* ^2^ = 33%). Another 11 study, in 373 patients with ATA, 227 women suffered ≥ 2 consecutive abortions, data from these studies determine the risk for recurrent miscarriage rate in relation to ATA (fixed‐effects OR = 2.34; 95% CI, 1.70–3.22; *P* < .00001; Figure 5), with moderate heterogeneity (*I* ^2^ = 44%).

### 3.3 TSH level in RM patients

**Figure 6:**
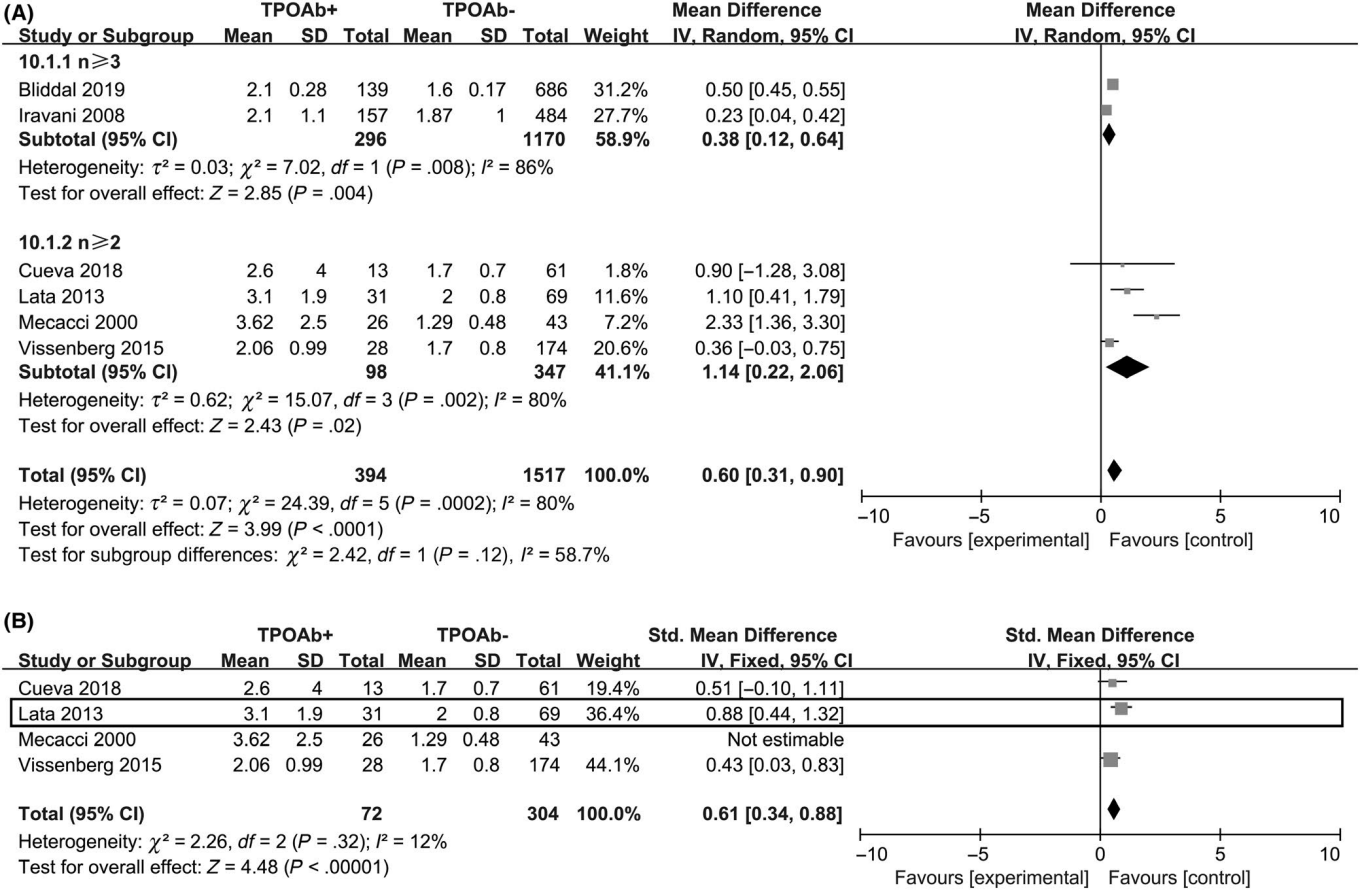
(A) Forest plot of TSH level in women with RM (n ≥ 3) or (n ≥ 2) comparing TPO‐Ab+ and TPO‐Ab− women; (B) Forest plot of TSH level in women with RM (n ≥ 2) comparing TPO‐Ab+ and TPO‐Ab− women, without the study of Mecacci. TPO‐Ab+ = positive for thyroid peroxidase; TPO‐Ab− = negative for thyroid peroxidase

We examined the difference of mean basal serum TSH between the TPO‐Ab‐positive and TPO‐Ab‐negative group. Six studies compared it with and without TPO‐Ab.^2^, ^17^, ^41^, ^47^, ^48^, ^49^ From the meta‐analysis, it emerged that TPO‐Ab‐positive women had significantly higher serum TSH levels (random‐effect SMD = 0.60; 95% CI, 0.31 to 0.90; *P* < .0001; Figure 6A). The *I* ^2^ value was 80% indicating the present of heterogeneity. Serum TSH concentrations were significantly increased in a study^47^. However, the subgroup pooled result changed when the study by Federico Mecacci^47^ was removed from the meta‐analysis (Fixed effect SMD = 0.60; 95% CI, 0.34 to 0.88; *P* < .00001; Figure 6B). When excluding the study by Mecacci,^47^ the combined results of n ≥ 2 subgroup showed mild heterogeneity (*I* ^2^ = 12%).

### 3.4 Live birth rate

**Figure 7:**
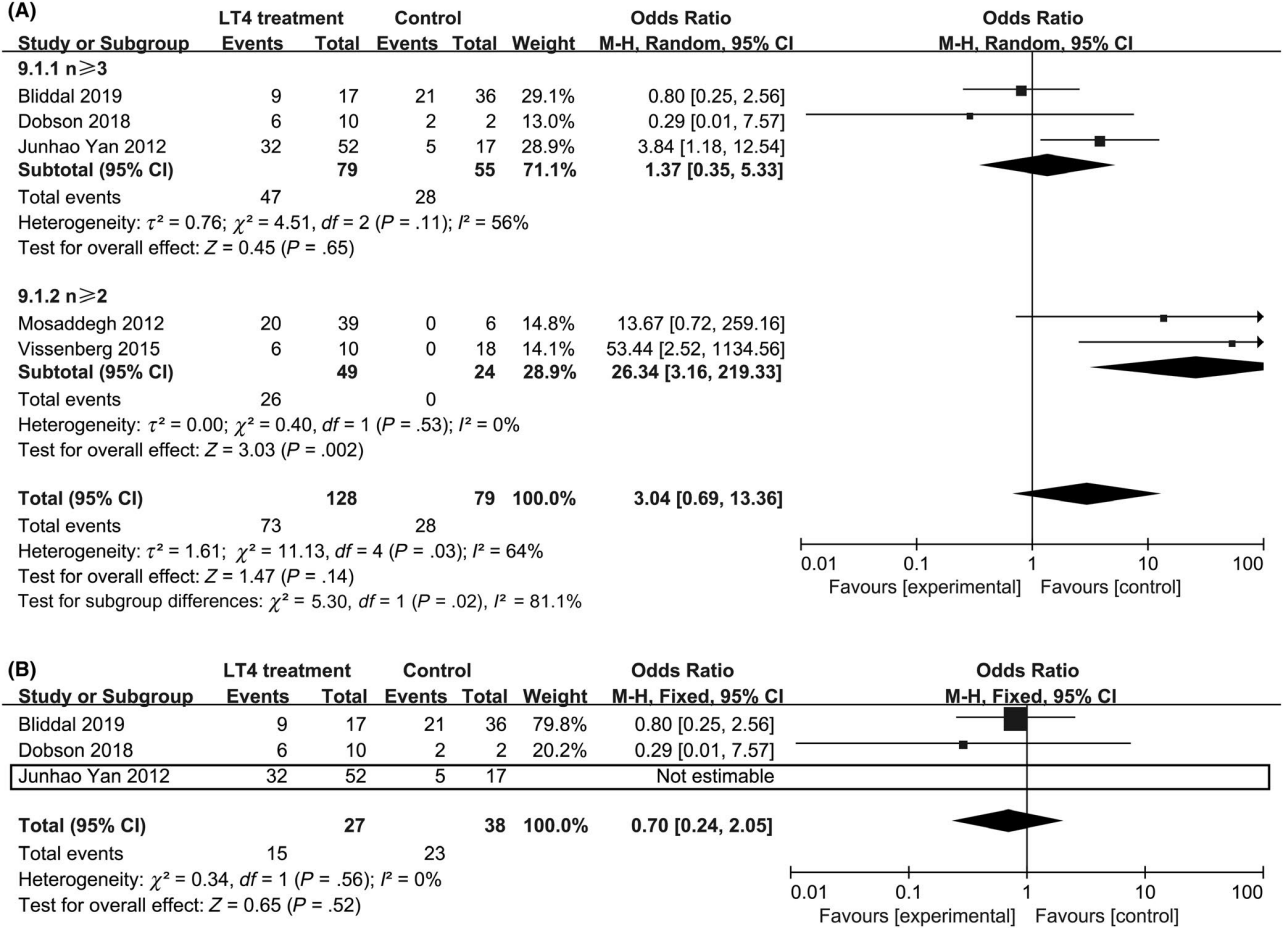
Forest plot of live birth rate, comparing TPO‐Ab+ and TPO‐Ab− women. TPO‐Ab+ = positive for thyroid peroxidase; TPO‐Ab− = negative for thyroid peroxidase

Five studies reported data on the association between LT4 supplementation and LBR in patients with TPO‐Ab. Three studies reported increased live birth rates among women receiving LT4 treatment,^2^, ^26^, ^50^ whereas the other two did not.^48^, ^51^ The combined results of all five studies indicated significant effect of LT4 treatment on the live birth rate, with a pooled OR of 3.04 (95% CI: 0.69‐13.36, n = 207, *I* ^2^ = 64%) (Figure 7A). When excluding the study by Junhao Yan,^50^ the subgroup combined results showed no significant effect of LT4 treatment on the live birth rate (OR = 0.70, 95% CI: 0.24‐2.05, n = 65, *I* ^2^ = 0%). But the I^2^ value was 0% indicating the absence of heterogeneity, a funnel plot showed no indication of asymmetry among studies.

## 4 DISCUSSION

The present systematic review and meta‐analysis showed clear evidence for a relationship between the presence of TPO‐Ab or ATA and the prevalence of RM. RM women with TPO‐Ab had higher TSH level when compared with those negative for TPO‐Ab. We also found beneficial effects of LT4 supplementation on the outcome of live birth rate among pregnant women with TPO‐Ab. The subgroup analysis further indicated that when women suffered ≥ 2 consecutive abortions, LT4 supplementation can effectively increase live birth rate. As these subgroup analyses were based on a limited number of studies, further research is needed to draw firm conclusions.

Based on evidence regarding the dynamics of TPO‐Ab, ATA and TSH levels during pregnancy,^52^ and the data (Figure 2) showed a significant heterogeneity from the study of Esplin.^34^ So we excluded the study of Esplin,^34^ from which samples were obtained ≥ 6 months after a pregnancy while the samples from other studies had been drawn no later than gestational week 8 or no pregnancy.

There has been found that women with TA has higher prevalence of recurrent miscarriage.^41^ Our contrasting results on prevalence rate in women with thyroid autoimmunity can be explained by several reasons. Among the 22 studies included in our meta‐analysis, one showed lower prevalence rate in TPO‐Ab positive women.^40^ However, the sample size of this study was relatively small. Additionally, the number of TPO‐Ab or ATA‐positive women enrolled in the studies was comparatively less than TPO‐Ab or ATA‐negative women in general.

Therefore, it has been suggested that thyroid auto antibodies may be employed as a marker for at‐risk pregnancies.^53^ Although the mechanism is not completely understood, it is postulated that TAI results in early pregnancy loss because of activation of immune system^54^ or act as an infertility factor and results in delayed conception.^55^

In this meta‐analysis, subjects with thyroid dysfunction or undergoing treatment were excluded in all the studies included. Although the TSH values in TPO‐Ab positive women were significantly higher than that in TPO‐Ab negative women. In particular, mean TSH was significantly higher by 0.60 mIU/L (95% CI 0.31‐0.90; *P* < .0001) in the TPO‐Ab‐positive group compared with TPO‐Ab‐negative group. The mean TSH of the participants in half studies was within the normal range (<2.5 mIU/L). Iravani^17^ excluded some participants who had serum TSH levels outside the reference range. In the other studies (Mecacci 2000,^47^ Lata 2013,^41^ Curve^49^), the TSH level was significantly higher than normal (>2.5 mIU/L). The data from Curve^49^ included some subclinical hypothyroidism patients.

Many institutions define RM as “the loss of three or more consecutive pregnancies”,^12^, ^56^ and biochemical pregnancies are included by The UK Royal College of Obstetricians and Gynaecologists (RCOG). While the American Society for Reproductive Medicine (ASRM) defines RM as “two or more failed clinical pregnancies”^13^ and exclude biochemical pregnancies. This rationale is supported by a large study with >1000 participants, which found that the likelihood of detecting an abnormality after two losses was similar to that after three or four or more losses.^57^ In our data, no matter how the definition changes, women with TPO‐Ab or ATA have a higher risk of RM than that in TPO‐Ab or ATA‐negative women.

Van den Boogard^24^ et al did a meta‐analysis in 2011, in which they found a clear evidence for a relationship between the presence of thyroid antibodies or subclinical hypothyroidism on unexplained subfertility, miscarriage, recurrent miscarriage, preterm birth, and post‐partum thyroid disease. But they paid no attention to treatment. Several meta‐analyses of studies of ART patients with higher levels of TPO‐Ab showed that substitution of thyroid hormones decreased the rate of miscarriages,^10^, ^58^, ^59^ and increased the delivery, clinical pregnancy, and fertilization rates.^60^ However, other studies such as the study by Wang published in 2017^27^ were unable to demonstrate the effect.

Live birth rate is an effective indicator for assessing pregnancy outcome. In the present meta‐analysis, we included more recently published studies and found a relative increment in live birth rate by LT4 supplementation among pregnant women with TPO‐Ab compared with that by no treatment/placebo. Subjects with thyroid dysfunction or undergoing treatment were excluded or analyzed separately in all the studies included.

Two studies^48^, ^51^ reported no significant increment in the LBR by LT4 supplementation, which is in contrast to the results^2^, ^26^, ^50^ for naturally conceiving women with TPO‐Ab who were likely to exhibit reduced PBR due to LT4 supplementation. But all of these studies never explain the type of conception. And Junhao Yan^50^ had different result from Dobson^51^ and Bliddal,^48^ considering its weight, we should sight the pooled result of three studies (OR = 1.37, 95% CI: 0.35‐5.33, n = 134, *P* = .65, *I* ^2^ = 56%). And the combined results of all five studies indicated significant effect of LT4 treatment on the live birth rate, with a pooled OR of 3.04 (95% CI: 0.69‐13.36, n = 207, *I* ^2^ = 64%). But in Lata's study,^41^ 31 patients with TPO‐Ab + were treated with 25 μg LT4, after that there was no difference in prevalence of miscarriage between hypothyroid and euthyroid individuals in TPO‐Ab + women.

Generally speaking, treatment for hypothyroidism usually begins with thyroxine (T4) replacement initiated at 25‐50 μg per day for 4 weeks and then verified according to biochemical and clinical analyses, this takes on average 6‐8 weeks. The dose is approximately 1.5 μg/body weight in pounds.^61^ In Mosaddegh's study,^26^ levothyroxine was used to TPO‐Ab + women, researchers vary considerably in TPO‐Ab level in different people. It was 25‐100 μg every day. This study showed that levothyroxine reduces the incidence of spontaneous abortions in women with high TPO‐Ab. It also decreased TPO‐Ab levels after 2‐3 months treatment. Therefore, further research is needed to draw firm conclusions, especially sighting the adjustment of medication orders on the basis of TPO‐Ab level.

Endometrial volume is an important parameter to evaluate endometrial receptivity and therefore a possible predictor for successful implantation.^62^, ^63^ Zhong et al reported lower implantation rates in women with TAI, but the authors did not report on thyroid function.^64^ The study of Merhan Dorostghoal^65^ demonstrate that the endometrial ER‐α expression may lead to defects in uterine receptivity and contribute to unexplained infertility. Furthermore, Zhangbi Wu^66^ indicated that TPO‐Ab induces a non‐receptive endometrial milieu in the euthyroid state, which may underlie the detrimental effects of Hashimoto's thyroiditis (HT) itself on embryo implantation. More research is needed to identify the role of thyroid autoantibodies on implantation.

Several studies have assessed the effect of different treatments of TPO‐Ab + women on pregnancy procedures. Thangaratinam et al pooled the results of two studies^67^, ^68^ and observed a significant reduction in risk of miscarriage in women treated with levothyroxine.^10^ Revelli et al retrospectively analyzed the effect of adjuvant treatments on IVF results in TAI + patients and they found that treating TAI + women with a combination of levothyroxine, acetylsalicylic acid and prednisolone resulted in higher ovarian responsiveness to gonadotropins and higher pregnancy rates but did not decrease the miscarriage rate.^4^ As TAI is hypothesized to be a marker of an underlying generalized autoimmune imbalance, Litwicka et al tested the efficacy of glucocorticoids administered alone and observed significantly higher clinical pregnancy and live birth rates in the treated group.^69^ But their sample size was small and all patients were treated in the same center.^69^ Vaquero et al compared intravenous immunoglobulin (IVIg) therapy and levothyroxine (LT4) replacement therapy for TAI during pregnancy and concluded that the abortion rate in the LT4 replacement group was significantly lower than in the IVIg group.^70^ In contrast, Sher et al compared the effect of heparin/aspirin therapy alone versus heparin/aspirin in combination with intravenous IVIg immunotherapy on IVF outcomes of patients with positive ATA.^71^ They found that IVIg was linked to increased live birth rate, but had no effect on miscarriage rate.^71^ In the meta‐analysis by Velkeniers et al, the authors concluded that LT4 treatment significantly improves delivery rate and reduces miscarriage rate in women undergoing ART, especially if the serum TSH level is ≥ 2.5 mIU/L with TAI or ≥4.0 mIU/L in general.^72^ In any case, all these studies followed different protocols and their results cannot be generalized. Further large scale studies regarding the cost effectiveness and safety profile of any treatment strategies are essential before we can establish the need for thyroid autoantibodies screening tests and treatments in patients with RM.

Our systematic review and meta‐analysis elucidates the association between thyroid autoimmunity and the prevalence of RM. The presence of thyroid autoantibodies may increase the TSH level in RM patients. This may recommend women to test for TPO‐Ab and TSH level after two pregnancy losses.

Antithyroid antibodies are known to occur in normal, healthy populations, and these autoantibodies are five times more common in women than in men.^73^ The clear association between TAI and RM suggests that women with RM need to be aggressively tested or treated for antithyroid antibodies. The tests and treatments are not only expensive but also involve potential health risks for the mother as well as the offspring. A recent study evaluated the association of maternal thyroid function during early pregnancy with offspring intelligence quotient and brain morphology in childhood and raised some concerns on possible overtreatment of patients with thyroxine, because offspring of patients with suppressed TSH may have worse neuropsychological outcomes.^44^ So we must concern proper screening crowd.

The statement from the American Society for Reproductive Medicine mentioned that the available data support the routine measurement of TSH in infertile women attempting pregnancy, but not that of TPO‐abs, unless TSH levels are ≥2.5 mIU/L.^74^ Nonetheless, TAI should not be neglected in women of childbearing age as it is a risk factor for thyroid dysfunction and hypothyroidism during pregnancy, and can affect fetal growth and neuropsychological outcome.^21^, ^75^ TAI can affect the pregnancy outcome in ART if elevated TSH levels are recorded simultaneously.^76^ Recently, the American Thyroid Association issued clinical practice guidelines after evaluating the evidence on thyroid function during ovarian stimulation.^77^ According to their guidelines, LT4 administration is recommended in women with TAI and TSH concentrations higher than the pregnancy‐specific reference range, whereas it may be considered in women without TAI and TSH concentrations higher than the pregnancy‐ specific reference range, but below 10 mIU/L.^78^ We propose that TSH levels of TAI women are monitored stringently for women with RM before pregnancy and further large scale prospective, randomized, placebo controlled trials are carried out to evaluate the effect of treatment for antithyroid antibodies in euthyroid women with RM.

It should be noted that our meta‐analysis had certain limitations. Due to the small number of studies included to analyze certain outcomes, we cannot rule out the existence of publication bias in our analysis.^79^ Furthermore, the included studies used different threshold values for TAI positivity (Table 1). 18 studies only measured TPO‐Ab as opposed to the remaining 11 studies which measured both TPO‐Ab and Tg‐Ab. It has been shown that women with TPO‐Ab + women had higher mean serum TSH levels than women without TPO‐Ab. Therefore, if we cannot use TPO‐Ab effectively evaluating the thyroid function of women with RM, we can test for TSH first.

Women with TAI are prone to develop subclinical hypothyroidism (SCH) during pregnancy, even though they can be euthyroid during the first trimester of pregnancy.^80^ Consequently, in order to decipher the effect of TSH on pregnancy outcome in women with RM, it is imperative to conduct further studies.

Our analysis opens the way for more fundamental studies in order to gain deeper insights into the pathophysiological mechanisms of thyroid autoimmunity. The supposed association between TAI and endometrium deserves attention in particular. The process of fertilization in TAI‐positive women also needs to be studied in details. Finally, we require further evidence regarding the factors involved in implantation of the embryo, including studies on endometrial receptivity, embryo quality and immunological factors.

## 5 CONCLUSION

This study reveals the association between thyroid autoimmunity per se and the prevalence of RM. Euthyroid women positive for antithyroid antibodies have higher risk of RM. TSH level in positive‐TPO‐Ab women with RM is higher than negative‐TPO‐Ab women. And LT4 supplementation may effectively increase live birth rate. More trials involving endometrial receptivity in TAI women should be carried out to decode the effect of TPO‐Ab or ATA. More RCTs are necessary to clarify the efficacy of LT4 in the treatment of RM. Advanced research is also necessary to elucidate the pathophysiological mechanisms of thyroid autoimmunity and its involvement in RM and subsequent pregnancy.

## CONFLICT OF INTEREST

The authors declare that there is no conflict of interest regarding the publication of this article.
